# Supplementary material for: Metacarpophalangeal Joint Pathology and Bone Mineral Density Increase with Exercise but Not with Incidence of Proximal Sesamoid Bone Fracture in Thoroughbred Racehorses
Source: Animals (Basel). 2023 Feb 24;13(5):827. doi: 10.3390/ani13050827 (PMC10000193; doi:10.3390/ani13050827)
Supplement: Supplementary file 1 [file animals-13-00827-s001.zip › Supplemental File S1.pdf]

Supplemental File S1: Details of case (unilateral PSB fracture) and control Thoroughbred racehorses

Table S1A: PSB Fracture

Horse ID	Sex	Age at death (years)	Age at start of career (years)	Total furlongs¶	Number of races	Career duration (weeks)
1†	F	2	2‡	0	0	0
2	F	2	2‡	48	0	7
3†	F	2	2‡	33	0	13
4†	M	3	2	63	3	23
5†	M	3	2	126	6	35
6	F	3	2	132	6	52
7†	F	3	2	183	5	57
8	MC	3	2	197	17	71
9†	F	4	2	197	9	94
10	M	4	2	349	13	99
11†	F	4	2	278	18	103
12	F	5	2	452	28	171
13	MC	5	2§	314	22	172
14†	MC	6	2	384	20	193
**Range**		2-6	2	0-384	0-28	0-193
**Median**		3	2	190	7.5	64

Table S1B: Control						
Horse ID	Sex	Age at death (years)	Age at start of career (years)	Total furlongs¶	Number of races	Career duration (weeks)
15†	F	2	2	36	1	12
16	M	2	2	40	3	13
17	F	2	2	42	3	17
18	M	3	2	54	1	25
19†	M	3	2§	142	8	38
20†	MC	3	2	136	3	56
21	MC	6	3	146	3	62
22†	MC	4	2§	131	5	69
23	MC	3	2	167	3	74
24†	MC	4	2	172	15	108
25†	F	4	2	268	11	117
26	F	4	2	300	12	130
27	F	5	2	375	19	169
28†	MC	5	2	517	32	179
29†	MC	6	2	520	40	212
**Range**		2-6	2-3	36-520	1-40	12-212
**Median**		4	2	146	5	69

Legend: F – Female, M – Male, MC – Male castrated, PSB – proximal sesamoid bone.
†Fracture contralateral (FXCL) or control contralateral (CLCL) PSB sectioned for Raman spectroscopy.
‡Horse started training at 2 years but never raced.
§Horse started training at 2 years but did not race until 3 years of age.
¶Total furlongs were rounded up to nearest whole number.
