# Supplementary material for: Metacarpophalangeal Joint Pathology and Bone Mineral Density Increase with Exercise but Not with Incidence of Proximal Sesamoid Bone Fracture in Thoroughbred Racehorses
Source: Animals (Basel). 2023 Feb 24;13(5):827. doi: 10.3390/ani13050827 (PMC10000193; doi:10.3390/ani13050827)
Supplement: Supplementary file 1 [file animals-13-00827-s001.zip › Supplemental File S2.pdf]

Supplemental File S2: Manual CT Measurement Descriptions

Metacarpal III

Qualitative Measurements

**Measurement**	**Grade**	**Criteria**
Medial or lateral subchondral (SC) bone irregularity	0	Normal
	1	Questionable flattening
	2	Flattening
Sagittal ridge SC bone irregularity	0	Normal
	1	Questionable flattening
	2	Flattening
SC cyst like lesions	0	Absent
	1	Focal indentation
	2	Cyst (expansion of size from the level of the isthmus)
Fracture	0	Absent
	1	Present
Medial or lateral parasagittal ridge lesion	0	No abnormality
	1	Lysis
	2	Incomplete
	3	Fracture
Cavitation of dorso-distal MC3	0	Absent
	1	Present
Fragmentation of dorso-distal MC3	0	Absent
	1	Present
Previous implants	0	Absent
	1	Present
Osteophytes of medial or lateral margin	0	Absent
	1	<1 mm
	2	1-2 mm
	3	>2 mm
SC sclerosis of lateral and medial 1/3 of condyle	0	Absent
	1	Present
Supracondylar lysis of lateral and medial 1/3 of condyle	0	Absent
	1	Present

Quantitative Measurements

<table>
<tr><th>Measurement</th><th></th></tr>
<tr><td rowspan="7">Max thickness Subchondral bone + sclerosis</td><td>Cross sectional area of sclerosis</td></tr>
<tr><td>Average HU of sclerosis</td></tr>
<tr><td>Standard deviation of sclerosis</td></tr>
<tr><td>Cross sectional area of condyle</td></tr>
<tr><td>Average HU of condyle</td></tr>
<tr><td>Standard deviation of condyle</td></tr>
<tr><td>Ratio of sclerosis to condyle</td></tr>
</table>

Proximal Sesamoid Bone

Qualitative Measurements

<table>
<tr><th>Measurement</th><th>Grade</th><th>Criteria</th></tr>
<tr><td rowspan="2">Area of necrosis/osteoporosis/osteopenia</td><td>0</td><td>Absent</td></tr>
<tr><td>1</td><td>Present</td></tr>
<tr><td rowspan="2">Fracture</td><td>0</td><td>Absent</td></tr>
<tr><td>1</td><td>Present</td></tr>
<tr><td rowspan="2">Osteophytes on apex</td><td>0</td><td>Absent</td></tr>
<tr><td>1</td><td><1 mm</td></tr>
<tr><td rowspan="2">Fracture</td><td>2</td><td>1-2 mm</td></tr>
<tr><td>3</td><td>>2 mm</td></tr>
<tr><td rowspan="4">Osteophytes on base</td><td>0</td><td>Absent</td></tr>
<tr><td>1</td><td><1 mm</td></tr>
<tr><td>2</td><td>1-2 mm</td></tr>
<tr><td>3</td><td>>2 mm</td></tr>
<tr><td rowspan="3">SCB irregularity
Enthesopathy of suspensory ligament</td><td>0</td><td>Normal</td></tr>
<tr><td>1</td><td>Mild irregularity</td></tr>
<tr><td>2</td><td>Severe irregularity</td></tr>
<tr><td rowspan="2">SCB irregularity
Sclerosis</td><td>0</td><td>Absent</td></tr>
<tr><td>1</td><td>Present</td></tr>
</table>

Quantitative Measurements

<table>
<tr><th>Measurement</th><th></th></tr>
<tr><td rowspan="3">Sagittal plane</td><td>Cross sectional area</td></tr>
<tr><td>Average HU of area</td></tr>
<tr><td>Standard deviation HU of area</td></tr>
<tr><td>Apical</td><td>Cross sectional area</td></tr>
</table>

	Average HU of area
	Standard deviation HU of area
Mid-body	Cross sectional area
	Average HU of area
	Standard deviation HU of area
Basilar	Cross sectional area
	Average HU of area
	Standard deviation HU of area
Subchondral	Cross sectional area
	Average HU of area
	SD HU of area
Flexor	Cross sectional area
	Average HU of area
	SD HU of area
Whole bone	Maximum height
	Maximum width
	Maximum depth

Proximal Phalanx I

Quantitative Measurements

**Measurement**	**Grade**	**Criteria**
Sagittal groove lysis	0	Absent
	1	Present
Osteophytosis dorsal	0	Absent
	1	<1 mm
Sagittal groove lysis	2	1-2 mm
	3	>2 mm
Osteophytosis medial or lateral	0	Absent
	1	<1 mm
	2	1-2 mm
	3	>2 mm
Dorsoproximal P1 chip fracture	0	Absent
	1	Present
Palmaroproximal P1 chip fracture	0	Absent
	1	Present

Qualitative Measurements

Measurement		
Dorsal plane	Max vascular channel diameter (mm)	
Sagittal plane	SC max bone plate thickness	Medial 1/3
		Lateral 1/3
		Sagittal groove
