# Supplementary material for: Metacarpophalangeal Joint Pathology and Bone Mineral Density Increase with Exercise but Not with Incidence of Proximal Sesamoid Bone Fracture in Thoroughbred Racehorses
Source: Animals (Basel). 2023 Feb 24;13(5):827. doi: 10.3390/ani13050827 (PMC10000193; doi:10.3390/ani13050827)
Supplement: Supplementary file 1 [file animals-13-00827-s001.zip › Supplemental File S3.pdf]

Supplement File S3: Raman Peak Fitting Methods

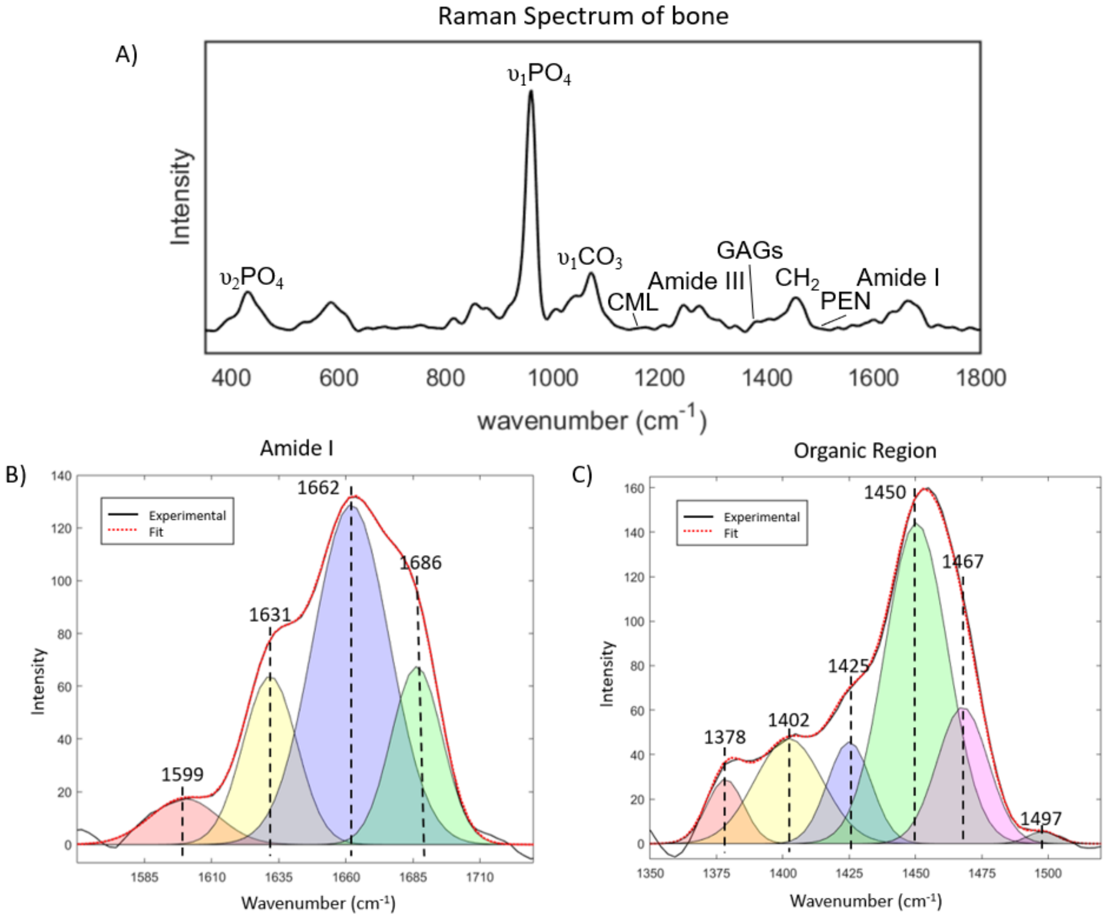

Figure S1 (A) Representative Raman spectrum of bone. Panels (B) and (C) show the resolved underlying bands obtained through second-derivative spectroscopy and curve-fitting analysis. The position of each sub-band is indicated by dashed vertical lines B) Spectrum of Amide I region (1545-1755 cm^{-1}). The integrated blue area indicates the relative content of the mature enzymatic trivalent crosslink Pyridinoline (PYD), and the green area indicates the relative content of the immature enzymatic divalent crosslink dehydrodihydroxylysinonorleucine (de-DHLNL). C) Spectrum of organic region (1350-1530 cm^{-1}). The integrated red area indicates the relative content of Glycosaminoglycans (GAGs), the green area indicates to relative content of CH2, and the grey area indicates the relative content of pentosidine.

Table S1: Raman imaging outcomes. Integration ranges for each band are listed in Table 2S

*Indicates intensity ratio resolved through second derivative spectroscopy and peak fitting

Raman Compositional Parameter	Description	Method of Analysis
Mineral:Matrix Ratio (MMR)	extent of mineralization of collagen matrix [1–3]	Integrated area ratio of v_2PO_4 to Amide III
Carbonate:Phosphate Ratio (CP)	extent of B-type carbonate substitution into mineral lattice [4,5]	Integrated area ratio of v_1CO_3 to v_2PO_4 band
Mineral Maturity/ Crystallinity (MMC)	Measure of crystal size and structural/ stoichiometric perfection [6]	the inverse of the full width at half-maximum intensity of the v_1PO_4 peak
Collagen Maturity (XLR)*	Relative ratio of mature trivalent to immature divalent enzymatic crosslinks in collagen matrix [7]	The ratio of the integrated areas of Amide I subbands representing enzymatic trivalent crosslinks and divalent crosslinks
Glycosaminoglycans (GAGs)*	Relative GAG content normalized to total organic matrix [8]	Integrated area ratio of CH_3 band to Amide III band
Pentosidine (PEN)*	Relative content of the AGE pentosidine [9,10]	Integrated area ratio of 1495cm^{-1} band to methylene side chains CH_2 band, the organic matrix representing both collagen and non-collagenous moieties of bone
Carboxymethyl-lysine (CML)	Relative content of the AGE Carboxymethyl-lysine [11,12]	Integrated area ratio of 1150 cm^{-1} to methylene side chains CH_2 band, the organic matrix representing both collagen and non-collagenous moieties of bone

Table S2: Primary band assignments in the Raman spectrum of bone

Vibrational Mode	Underlying Sub-band	Peak Position/ Integration Range (cm^{-1})	Sub-band Position (cm^{-1})	Comments	References
		410-460			[13–15]
$\nu_2PO_4^{3-}$		430			[13,14]
	$\nu_2PO_4^{3-}$ shoulder		450		[13,14]
		930-980			[16]
$\nu_1PO_4^{2-}$	$\nu_1PO_4^{2-}$		960	Pure P-O stretching	[13,17]
	$\nu_1PO_4^{2-}$		955	Immature bone mineral	[13,17]
	$\nu_1PO_4^{2-}$		957-962	Mature bone mineral/ carbonate apatite	[13,17]
		1050-1100			[13,18]
$\nu_1CO_3^{2-}$	$\nu_1CO_3^{2-}$-$\nu_3PO_4^{2-}$		1030	Contains overlap from asymmetric stretching $\nu_3PO_4^{3-}$	[13]
	$\nu_1CO_3^{2-}$-$\nu_3PO_4^{2-}$		1046	Contains overlap from asymmetric stretching $\nu_3PO_4^{3-}$	[13]
	$\nu_3PO_4^{2-}$		1076		[13,18]
		1150			[19]
$\nu(NC)+\tau(HCCO)$	$\nu(NC)+\tau(HCCO)$		1145	Contributions from $\nu(NC)+\tau(HCCO)$ are different	[19]
	$\nu(NC)+\tau(HCCO)$		1156	Contributions from $\nu(NC)+\tau(HCCO)$ are different	[19]
		1215-1300			[13,15,20]
Amide III			1242	Doublet peak	[13,20]
			1270		[13,20]
δCH_3		1365-1390		Predominantly symmetric deformation δCH_3 (GAGs)	[15,21]
δCH_2		1446		δCH_2, contains contributions from proteins	[13,14]
PEN		1495			[22,23]
		1620-1700			[13,16]
	$\delta(C{=}C)$		1609		[13]
Amide I	$\nu(C{=}C)$		1640		[13]
	Pyridinoline		1660	Mature trivalent crosslinks	[13,24]
	de-DHLNL		1690	Immature divalent crosslinks	[13,24]

* Abbreviation: GAGs – Glycosaminoglycans; PEN – Pentosidine; de-DHLNL – dehydrodihydroxylysinonorleucine.

References

1. Kazanci, M., Roschger, P., Paschalis, E.P., Klaushofer, K. and Fratzl, P. (2006) Bone osteonal tissues by Raman spectral mapping: Orientation-composition. *Journal of Structural Biology* **156**, 489–496.
2. Roschger, A., Gamsjaeger, S., Hofstetter, B., Masic, A., Blouin, S., Messmer, P., Berzlanovich, A., Paschalis, E.P., Roschger, P., Klaushofer, K. and Fratzl, P. (2014) Relationship between the v2PO4/amide III ratio assessed by Raman spectroscopy and the calcium content measured by quantitative backscattered electron microscopy in healthy human osteonal bone. *Journal of Biomedical Optics* **19**, 065002.
3. Taylor, E.A., Lloyd, A.A., Salazar-Lara, C. and Donnelly, E. (2017) Raman and Fourier Transform Infrared (FT-IR) Mineral to Matrix Ratios Correlate with Physical Chemical Properties of Model Compounds and Native Bone Tissue. *Applied Spectroscopy* **71**, 2404–2410.
4. Penel, G., Leroy, G., Rey, C. and Bres, E. (1998) MicroRaman spectral study of the PO4 and CO3 vibrational modes in synthetic and biological apatites. *Calcified Tissue International* **63**, 475–481.
5. Awonusi, A., Morris, M.D. and Tecklenburg, M.M.J. (2007) Carbonate assignment and calibration in the Raman spectrum of apatite. *Calcified Tissue International* **81**, 46–52.
6. Kazanci, M., Fratzl, P., Klaushofer, K. and Paschalis, E.P. (2006) Complementary information on in vitro conversion of amorphous (precursor) calcium phosphate to hydroxyapatite from raman microspectroscopy and wide-angle X-ray scattering. *Calcified Tissue International* **79**, 354–359.
7. Gamsjaeger, S., Robins, S.P., Tatakis, D.N., Klaushofer, K. and Paschalis, E.P. (2017) Identification of Pyridinoline Trivalent Collagen Cross-Links by Raman Microspectroscopy. *Calcified Tissue International* **100**, 565–574.
8. Gamsjaeger, S., Klaushofer, K. and Paschalis, E.P. (2014) Raman analysis of proteoglycans simultaneously in bone and cartilage. *Journal of Raman Spectroscopy* **45**, 794–800.
9. Rokidi, S., Paschalis, E.P., Klaushofer, K., Vennin, S., Desyatova, A., Turner, J.A., Watson, P., Lappe, J., Akhter, M.P. and Recker, R.R. (2019) Organic matrix quality discriminates between age- and BMD-matched fracturing versus non-fracturing post-menopausal women: A pilot study. *Bone* **127**, 207–214.
10. Gamsjaeger, S., Srivastava, A.K., Wergedal, J.E., Zwerina, J., Klaushofer, K., Paschalis, E.P. and Tatakis, D.N. (2014) Altered bone material properties in HLA-B27 rats include reduced mineral to matrix ratio and altered collagen cross-links. *Journal of Bone and Mineral Research* **29**, 2382–2391.
11. Pawlak, A.M., Beattie, J.R., Glenn, J. V., Stitt, A.W. and McGarvey, J.J. (2008) Raman spectroscopy of advanced glycation end products (AGEs), possible markers for progressive retinal dysfunction. *Journal of Raman Spectroscopy* **39**, 1635–1642.
12. Rubin, M.R., Paschalis, E.P., Poundarik, A., Sroga, G.E., McMahon, D.J., Gamsjaeger, S., Klaushofer, K. and Vashishth, D. (2016) Advanced glycation endproducts and bone material properties in type 1 diabetic mice. *PLoS ONE* **11**, 1–14.
13. Mandair, G.S. and Morris, M.D. (2015) Contributions of Raman spectroscopy to the understanding of bone strength. *BoneKEy reports* **4**. https://pubmed.ncbi.nlm.nih.gov/25628882/. Accessed March 14, 2022.
14. Penel, G., Delfosse, C., Descamps, M. and Leroy, G. (2005) Composition of bone and apatitic biomaterials as revealed by intravital Raman microspectroscopy. *Bone* **36**, 893–901. https://pubmed.ncbi.nlm.nih.gov/15814305/. Accessed March 14, 2022.

15. Paschalis, E.P., Gamsjaeger, S., Hassler, N., Klaushofer, K. and Burr, D. (2017) Ovarian hormone depletion affects cortical bone quality differently on different skeletal envelopes. *Bone* **95**, 55–64.
16. Rubin, M.R., Paschalis, E.P., Poundarik, A., Sroga, G.E., McMahon, D.J., Gamsjaeger, S., Klaushofer, K. and Vashishth, D. (2016) Advanced Glycation Endproducts and Bone Material Properties in Type 1 Diabetic Mice. *PLOS ONE* **11**, e0154700. https://journals.plos.org/plosone/article?id=10.1371/journal.pone.0154700. Accessed March 14, 2022.
17. Crane, N.J., Popescu, V., Morris, M.D., Steenhuis, P. and Ignelzi, M.A. (2006) Raman spectroscopic evidence for octacalcium phosphate and other transient mineral species deposited during intramembranous mineralization. *Bone* **39**, 434–442. https://pubmed.ncbi.nlm.nih.gov/16627026/. Accessed March 14, 2022.
18. Awonusi, A., Morris, M.D. and Tecklenburg, M.M.J. (2007) Carbonate assignment and calibration in the Raman spectrum of apatite. *Calcified tissue international* **81**, 46–52. https://pubmed.ncbi.nlm.nih.gov/17551767/. Accessed March 14, 2022.
19. Téllez S., C.A., Mendes, T.O., dos Santos, L., Silva, M.G.P., Pereira, L., Fávero, P., Singh, P. and Martin, A.A. (2019) Combined in vivo confocal Raman spectroscopy and density functional theory to detect carboxymethyl(lysine) in the human stratum corneum. *Vibrational Spectroscopy* **100**, 40–47.
20. Dehring, K.A., Crane, N.J., Smukler, A.R., McHugh, J.B., Roessler, B.J. and Morris, M.D. (2006) Identifying chemical changes in subchondral bone taken from murine knee joints using Raman spectroscopy. *Applied spectroscopy* **60**, 1134–1141. https://pubmed.ncbi.nlm.nih.gov/17059665/. Accessed March 14, 2022.
21. Gamsjaeger, S., Klaushofer, K. and Paschalis, E.P. (2014) Raman analysis of proteoglycans simultaneously in bone and cartilage. *Journal of Raman Spectroscopy* **45**, 794–800. https://onlinelibrary.wiley.com/doi/full/10.1002/jrs.4552. Accessed March 14, 2022.
22. Unal, M. and Akkus, O. (2015) Raman spectral classification of mineral- and collagen-bound water's associations to elastic and post-yield mechanical properties of cortical bone. *Bone* **81**, 315–326. https://pubmed.ncbi.nlm.nih.gov/26211992/. Accessed March 13, 2022.
23. Rokidi, S., Paschalis, E.P., Klaushofer, K., Vennin, S., Desyatova, A., Turner, J.A., Watson, P., Lappe, J., Akhter, M.P. and Recker, R.R. (2019) Organic matrix quality discriminates between age- and BMD-matched fracturing versus non-fracturing post-menopausal women: A pilot study. *Bone* **127**, 207–214. https://pubmed.ncbi.nlm.nih.gov/31229674/. Accessed March 14, 2022.
24. Gamsjaeger, S., Robins, S.P., Tatakis, D.N., Klaushofer, K. and Paschalis, E.P. (2017) Identification of Pyridinoline Trivalent Collagen Cross-Links by Raman Microspectroscopy. *Calcified tissue international* **100**, 565–574. https://pubmed.ncbi.nlm.nih.gov/28246932/. Accessed March 14, 2022.
