# Supplementary material for: Metacarpophalangeal Joint Pathology and Bone Mineral Density Increase with Exercise but Not with Incidence of Proximal Sesamoid Bone Fracture in Thoroughbred Racehorses
Source: Animals (Basel). 2023 Feb 24;13(5):827. doi: 10.3390/ani13050827 (PMC10000193; doi:10.3390/ani13050827)
Supplement: Supplementary file 1 [file animals-13-00827-s001.zip › Supplemental File S4.pdf]

Supplement File S4: Raman results table; p-value for all fixed effects (* p<0.05)

	Group	Sex	Total Furlongs	Region
**Mineral:Matrix Ratio**				
9 sub-regions	0.89	0.85	0.96	*1.59e-11**
3 sub-regions (dorsal-	0.88	0.84	0.96	*7.12e-08**
3 sub-regions (proximal-	0.89	0.83	0.95	*0.04**
**Carbonate:Phosphate**				
9 sub-regions	0.19	0.37	0.05	*0.005**
3 sub-regions (dorsal-	0.19	0.37	0.05	*0.0009**
3 sub-regions (proximal-	0.19	0.37	0.05	0.06
**Mineral**				
9 sub-regions	0.76	0.71	0.28	0.11
3 sub-regions (dorsal-	0.76	0.71	0.27	0.54
3 sub-regions (proximal-	0.75	0.69	0.27	0.31
**Collagen Maturity**				
9 sub-regions	0.39	0.25	0.84	0.05
3 sub-regions (dorsal-	0.39	0.25	0.84	0.10
3 sub-regions (proximal-	0.39	0.25	0.84	0.19
**Carboxymethyl-lysine**				
9 sub-regions	0.62	0.99	0.33	*0.01**
3 sub-regions (dorsal-	0.61	0.99	0.33	*0.01**
3 sub-regions (proximal-	0.62	1.00	0.33	0.74
**Pentosidine**				
9 sub-regions	0.99	0.13	0.41	*0.01**
3 sub-regions (dorsal-	1.00	0.14	0.43	*0.047**
3 sub-regions (proximal-	0.99	0.13	0.42	0.76
**Glycosaminoglycans**				
9 sub-regions	0.83	0.24	0.69	*0.004**
3 sub-regions (dorsal-	0.83	0.24	0.70	0.05
3 sub-regions (proximal-	0.82	0.24	0.69	0.55
