# Supplementary material for: Metacarpophalangeal Joint Pathology and Bone Mineral Density Increase with Exercise but Not with Incidence of Proximal Sesamoid Bone Fracture in Thoroughbred Racehorses
Source: Animals (Basel). 2023 Feb 24;13(5):827. doi: 10.3390/ani13050827 (PMC10000193; doi:10.3390/ani13050827)
Supplement: Supplementary file 1 [file animals-13-00827-s001.zip › Supplemental File S5.pdf]

Supplemental File S5: Multivariate correlation analysis between exercise variables and horse age

**Exercise history variable**	**Pearson's correlation coefficient (r)**	***P*-value**
Career duration (weeks)	0.88	*P*<0.0001
Total weeks rest	0.86	*P*<0.0001
Total weeks worked	0.84	*P*<0.0001
Total furlongs	0.83	*P*<0.0001
Total # races	0.75	*P*<0.0001
# of breaks ≥8 weeks no work	0.66	*P*=0.0002
Career work:rest ratio	-0.62	*P*=0.0006
