# Supplementary material for: Metacarpophalangeal Joint Pathology and Bone Mineral Density Increase with Exercise but Not with Incidence of Proximal Sesamoid Bone Fracture in Thoroughbred Racehorses
Source: Animals (Basel). 2023 Feb 24;13(5):827. doi: 10.3390/ani13050827 (PMC10000193; doi:10.3390/ani13050827)
Supplement: Supplementary file 1 [file animals-13-00827-s001.zip › Supplemental File S6.pdf]

Supplemental File S6: Computed tomography results table; p-value for all fixed effects (* p<0.05)

			P-value		
**Measurement**		ICC	Group	Sex	Total Furlong
**Third metacarpal bone (MC3)**					
1) Condyle subchondral bone irregularity					
a	Lateral (0=normal, 1=questionable flattening, 2=flattening)	1			
b	Medial (0=normal, 1=questionable flattening, 2=flattening)	1			
2) Sagittal ridge subchondral bone irregularity (0=normal, 1=questionable flattening, 2=flattening)		0.86	0.17	0.62	0.62
3) Subchondral cyst like lesions (0=absent, 1=focal lucency, 2=focal indent, 3=cyst)		0.86			
a	Location(s)				
4) Fracture (absent=0, present=1)		0.77			
5) Parasagittal ridge subchondral bone cyst like lesion					
a	Lateral (0=absent, 1=focal lucency, 2=focal indent, 3=cyst)	0.56	0.93	0.32	0.58
b	Medial (0=absent, 1=focal lucency, 2=focal indent, 3=cyst)	0.73	0.77	0.79	0.64
6) Supracondylar lysis (absent=0, present=1)					
a	Lateral (absent=0, present=1)	0.57			
b	Medial (absent=0, present=1)	0.59			
7) Cavitation of dorso-distal surface of MC3: (absent=0, present=1)		0.78	*0.0007**	0.43	0.11
8) Fragmentation of dorso-distal surface of MC3: (absent=0, present=1)		0.77	0.37	0.64	0.09
9) Enthesiophytosis of dorso-distal surface of MC3: (absent=0, present=1)		0.92	0.67	0.99	*0.01**
10) Previous implants (absent=0, present=1)		0.58			
11) Osteophytes on medial and/or lateral margins					
a	Lateral (absent=0, mild=1, severe=2)	0.69			
b	Medial (absent=0, mild=1, severe=2)	0.60			
**Lateral condyle**					
12) Subchondral sclerosis (absent=0, present=1)		1			
a	If yes, maximum thickness of subchondral bone+ sclerosis (mm, sagittal plane)	0.99	0.3	0.29	*<0.0001**
b	Cross sectional area of sclerosis (cm^2)	0.77	0.69	0.26	*0.0021**
c	Cross sectional area of sclerosis (mm^2)	0.76			
d	Avg HU of this area	1	0.53	0.80	0.68
e	SD HU of this area	0.50	0.57	0.32	0.19

f	Cross sectional area of condyle (cm^2)	1	0.0002*	0.0021*	0.027*
g	Cross sectional area of condyle (mm^2)	1	0.0003*	0.0029*	0.032*
h	Avg HU of this area	0.65	0.62	0.58	0.0001*
i	SD HU of this area	0.56	0.52	0.50	0.28
j	Ratio of sclerosis to condyle (area)		0.30	0.84	0.02*
k	Ratio of sclerosis to condyle (Avg of HU)		0.99	0.34	<0.0001*
l	Ratio of sclerosis to condyle (SD of HU)		0.69	0.27	0.03*
**Medial condyle**					
13) Subchondral sclerosis (absent=0, present=1)		1			
a	If yes, maximum thickness of subchondral bone+ sclerosis (mm, sagittal plane)	0.60			
b	Cross sectional area of sclerosis (cm^2)	1			
c	Cross sectional area of sclerosis (mm^2)	1	0.40	0.35	0.09*
d	Avg HU of this area	0.77	0.47	0.54	0.37
e	SD HU of this area	1	0.37	0.32	0.98
f	Cross sectional area of condyle (cm^2)	0.66	0.0033*	0.0018*	0.0022*
g	Cross sectional area of condyle (mm^2)	0.66	0.0033*	0.0018*	0.0022*
h	Avg HU of this area	0.67	0.42	0.78	0.81
i	SD HU of this area	0.56	0.52	0.52	0.48
j	Ratio of sclerosis to condyle (area)		0.25	0.92	0.19
k	Ratio of sclerosis to condyle (Avg of HU)	0.79	0.16	0.78	0.31
l	Ratio of sclerosis to condyle (SD of HU)	0.99			

	P-value			
**Measurement**	ICC	Group	Sex	Total Furlong
**Proximal sesamoid bone**				
**Lateral**				
14) Area of necrosis/ osteoporosis/osteopenia (absent=0, present=1)	0.77			
15) Max height (cm)	1	0.49	0.44	0.87
16) Max width (cm)	0.61	0.027*	0.12	0.24
17) Max depth (cm)	1	0.0067*	0.0067*	0.12
18) Fracture (absent=0, present=1)	0.97	<0.0001*	0.48	0.43
a Fracture configuration				
19) Osteophytes on apex (absent=0, mild=1, severe=2)	0.68	0.50	0.86	0.06*
20) Osteophytes on base Osteophytes on apex (absent=0, mild=1, severe=2)	0.83	0.74	0.75	0.06*

21) Subchondral bone irregularity (absent=0, mild=1, severe=2)		0.69	0.39	0.46	0.45
22) Enthesopathy					
a	Enthesopathy of suspensory branch (absent=0, present=1)	0.02			
b	Enthesopathy of DSL (absent=0, present=1)	0.74			
c	Enthesopathy of intersesamoidian ligament (absent=0, present=1)	0.73	0.03*	0.25	0.22
23) Sclerosis					
a	Subchondral (absent=0, present=1)	0.56			
b	Flexor (absent=0, present=1)	0.85			
c	Apical (absent=0, present=1)	0.60	0.98	0.43	0.49
24) Whole bone in sagittal plane					
a	Cross sectional area	0.86	0.04*	0.32	0.17
b	Avg HU of this area	0.68	0.38	0.23	0.01*
c	SD HU of this area	0.85	0.41	0.76	0.76
25) Apical					
a	Cross sectional area (cm^2)	0.73	0.20	0.64	
b	Avg HU of this area	0.68	0.17	0.34	0.024*
c	SD HU of this area	1	0.90	0.55	0.39
26) Midbody					
a	Cross sectional area	1	0.18	0.08	0.14
b	Avg HU of this area	0.77	0.62	0.005*	0.08
c	SD HU of this area	0.76	0.77	0.76	0.12
27) Basilar					
a	Cross sectional area	0.60	0.0002*	0.06	0.025*
b	Avg HU of this area		0.29	0.36	0.34
c	SD HU of this area	0.71	0.13	0.20	0.18
28) Subchondral					
a	Cross sectional area	0.76	0.22	0.50	0.39
b	Avg HU of this area	1	0.64	0.20	0.0006*
c	SD HU of this area	1	0.94	0.63	0.65
29) Midbody					
a	Cross sectional area	1	0.027*	0.027*	0.95
b	Avg HU of this area	0.68	0.51	0.01*	0.003*
c	SD HU of this area	0.72	0.95	0.36	0.59
30) Flexor					
a	Cross sectional area	0.75			
b	Avg HU of this area	0.78	0.21	0.28	0.022*
c	SD HU of this area	0.98	0.94	0.82	0.97
31) Axial					

a	Cross sectional area	1			
b	Avg HU of this area	0.61	0.48	0.14	_0.018*_
c	SD HU of this area	0.73	0.92	0.34	0.86
32) Mid-sagittal					
a	Cross sectional area	0.93	0.99	0.99	0.25
b	Avg HU of this area	0.78	0.70	_0.036*_	_0.05_
c	SD HU of this area	0.71	0.58	0.42	0.53
33) Abaxial					
a	Cross sectional area	1	0.55	0.76	_0.035*_
b	Avg HU of this area	0.65	0.08	0.49	0.51
c	SD HU of this area	0.94	0.36	0.34	_0.001*_
**Medial**					
34) Area of necrosis/ osteoporosis/osteopenia (absent=0, present=1)		0.68	0.64	0.47	0.43
35) Max height (cm)		0.74	_0.044*_	_0.030*_	0.23
36) Max width (cm)		0.85	0.86	_0.014*_	0.43
37) Max depth (cm)		1	0.70	0.69	0.08
38) Fracture (absent=0, present=1)			<0.0001*	0.48	0.43
a	Fracture configuration				
39) Osteophytes on apex (absent=0, mild=1, severe=2)		0.77	0.85	0.95	0.26
40) Osteophytes on base (absent=0, mild=1, severe=2)		0.60	0.09	0.52	_0.013*_
41) Subchondral bone irregularity (absent=0, mild=1, severe=2)		0.72			
42) Enthesopathy					
a	Enthesopathy of suspensory branch (absent=0, present=1)	0.60			
b	Enthesopathy of DSL (absent=0, present=1)	0.60	0.80	0.91	0.54
c	Enthesopathy of intersesamoidian ligament (absent=0, present=1)	0.77			
43) Sclerosis					
a	Subchondral (absent=0, present=1)	0.57	_0.039*_	0.26	0.25
b	Flexor (absent=0, present=1)	0.57	_0.039*_	0.26	0.25
c	Apical (absent=0, present=1)	0.60	0.99	0.52	_0.020*_
44) Whole bone in sagittal plane					
a	Cross sectional area	1	_0.004*_	0.48	_0.009*_
b	Avg HU of this area	0.79			
c	SD HU of this area	1	0.31	0.78	0.81
45) Apical					
a	Cross sectional area (cm^2)	0.69	0.80	0.82	0.60
b	Avg HU of this area	0.77	0.57	0.13	_0.002*_

c	SD HU of this area	0.73			
46) Midbody					
a	Cross sectional area	0.75	0.29	*0.003**	0.20
b	Avg HU of this area	0.68	0.26	0.09	0.24
c	SD HU of this area	1	0.47	0.57	0.38
47) Basilar					
a	Cross sectional area	1	*0.040**	0.23	0.26
b	Avg HU of this area	0.71			
c	SD HU of this area	0.85			
48) Subchondral					
a	Cross sectional area	0.77			
b	Avg HU of this area	0.72	0.53	*0.006**	*0.001**
c	SD HU of this area	0.73	0.52	0.53	0.26
49) Midbody					
a	Cross sectional area	0.94	0.18	*0.005**	0.15
b	Avg HU of this area	0.77			
c	SD HU of this area	1			
50) Flexor					
a	Cross sectional area	0.84	*0.035**	0.47	0.70
b	Avg HU of this area	0.77	0.90	0.23	0.11
c	SD HU of this area	1	0.07	0.92	0.20
51) Axial					
a	Cross sectional area	0.72	0.70	0.78	0.46
b	Avg HU of this area	0.77	0.19	*0.005**	*0.0004**
c	SD HU of this area	0.78			
52) Mid-sagittal					
a	Cross sectional area	0.68	0.23	0.62	*0.039**
b	Avg HU of this area	0.69	0.29	0.37	0.97
c	SD HU of this area	0.66	0.19	0.65	0.06
53) Abaxial					
a	Cross sectional area	0.88	0.08	0.87	0.12
b	Avg HU of this area	0.63	0.21	0.08	0.71
c	SD HU of this area	1	0.47	0.55	0.18

		P-value			
**Measurement**		ICC	Group	Sex	Total Furlong
**Proximal phalanx (P1)**					
54) Sagittal groove lysis (absent=0, present=1)		0.56	0.85		
55) Osteophytosis dorsal		0.98			
a	Osteophytosis dorsolaterally (absent=0, mild=1, severe=2)	0.73			
b	Osteophytosis dorsomedially (absent=0, mild=1, severe=2)	0.79			
56) Osteophytosis lateral or medial					
a	Osteophytosis lateral (absent=0, mild=1, severe=2)	0.78			
b	Osteophytosis medial (absent=0, mild=1, severe=2)	1	0.80	0.80	0.64
57) Dorsoproximal P1 chip Fx (absent=0, present=1)		0.79			
a	If present, record maximum dimension (mm)				
58) Palmaroproximal P1 chip Fx (absent=0, present=1)		0.99			
a	If present, record maximum dimension (mm)		0.51	0.68	0.28
59) Subchondral bone plate thickness (measured in sagittal plane)					
a	Medial 1/3 - max thickness (mm)	0.57			
b	Lateral 1/3 - max thickness (mm)	1			
c	Sagittal grove - max thickness (mm)	0.55	*0.004**	*0.0001**	0.78
60) Subchondral bone plate thickness (measured in frontal plane)					
d	Medial 1/3 - max thickness (mm)	0.70	0.67	1.00	0.07
e	Lateral 1/3 - max thickness (mm)	0.71			
f	Sagittal grove - max thickness (mm)	0.62	0.83	0.80	*<0.0001**
61) P1 subchondral bone irregularity (0=normal, 1=questionable flattening, 2=flattening)					
62) P1 subchondral bone cyst like lesion (0=absent, 1=focal lucency, 2=focal indent, 3=cyst)			0.64	*0.047**	0.98

Legend: ICC - intra-class correlation coefficient, Avg – average, SD – standard deviation, HU – Hounsfield units, PSB – proximal sesamoid bone, P1 – proximal phalanx, DSL – distal sesamoidean ligament, Fx – fracture
