# Supplementary material for: Metacarpophalangeal Joint Pathology and Bone Mineral Density Increase with Exercise but Not with Incidence of Proximal Sesamoid Bone Fracture in Thoroughbred Racehorses
Source: Animals (Basel). 2023 Feb 24;13(5):827. doi: 10.3390/ani13050827 (PMC10000193; doi:10.3390/ani13050827)
Supplement: Supplementary file 1 [file animals-13-00827-s001.zip › Supplemental File S7.pdf]

Supplemental File S7: DXA results table

Region	Group	Mean BMD g/cm^2	Standard deviation	P-value (Fracture vs Control)	P-value (Total furlongs)
Distal MC3	Fracture	2.99	0.20	0.86	<0.0001*
	Control	3.00	0.22		
	**Total**	**3.03**	**0.22**		
Palmar Distal MC3	Fracture	3.14	0.34	0.38	0.0001*
	Control	3.22	0.33		
	**Total**	**3.23**	**0.32**		
PSB	Fracture	2.13	0.07	0.64	0.06
	Control	2.10	0.11		
	**Total**	**2.12**	**0.10**		

Legend: MC3 – Metacarpal 3, PSB – Proximal Sesamoid Bone, BMD – Bone Mineral Density
