# Supplementary material for: Metacarpophalangeal Joint Pathology and Bone Mineral Density Increase with Exercise but Not with Incidence of Proximal Sesamoid Bone Fracture in Thoroughbred Racehorses
Source: Animals (Basel). 2023 Feb 24;13(5):827. doi: 10.3390/ani13050827 (PMC10000193; doi:10.3390/ani13050827)
Supplement: Supplementary file 1 [file animals-13-00827-s001.zip › Supplemental File S8.pdf]

Supplemental File S8: Ash fraction (reported as % mineral by weight) by region

Region	Group	Percent mineral by weight	Standard deviation	P-value (fx. vs control)	P-value (total furlongs)
Apical	Fracture	58.8	2.1	0.26	0.96
	Control	59.5	1.5		
	**Total**	**59.1**	**1.8**		
Mid-body	Fracture	61.0	1.6	0.36	0.07
	Control	61.5	0.7		
	**Total**	**61.3**	**1.2**		
Basilar	Fracture	59.7	1.7	0.09	0.47
	Control	60.8	1.1		
	**Total**	**60.3**	**1.5**		
Subchondral	Fracture	59.4	2.1	0.06	**0.04***
	Control	60.4	1.0		
	**Total**	**59.9**	**1.7**		
Medullary	Fracture	62.1	1.4	0.19	0.19
	Control	62.6	0.7		
	**Total**	**62.3**	**1.1**		
Flexor	Fracture	58.1	2.1	0.53	0.45
	Control	58.6	1.4		
	**Total**	**58.4**	**1.7**		
Whole PSB Bone	Fracture	59.8	1.6	0.16	0.40
	Control	60.6	0.8		
	**Total**	**60.2**	**1.3**		
Tuber Coxae	Fracture	53.0	4.6	0.94	0.43
	Control	53.7	3.3		
	**Total**	**53.4**	**3.9**		

The mean percent mineral (by weight) of whole PSBs, PSB subregions, and tuber coxae from fracture (n=14) and control (n=15) cases. The mid-body medullary sub-region was not included in percent mineral comparisons. Percent mineral of the subchondral region increased with total furlongs.
