# Supplementary material for: Metacarpophalangeal Joint Pathology and Bone Mineral Density Increase with Exercise but Not with Incidence of Proximal Sesamoid Bone Fracture in Thoroughbred Racehorses
Source: Animals (Basel). 2023 Feb 24;13(5):827. doi: 10.3390/ani13050827 (PMC10000193; doi:10.3390/ani13050827)
Supplement: Supplementary file 1 [file animals-13-00827-s001.zip › Supplemental File S9.pdf]

Supplemental File S9: Ash fraction (reported as % mineral by weight) regional comparison results

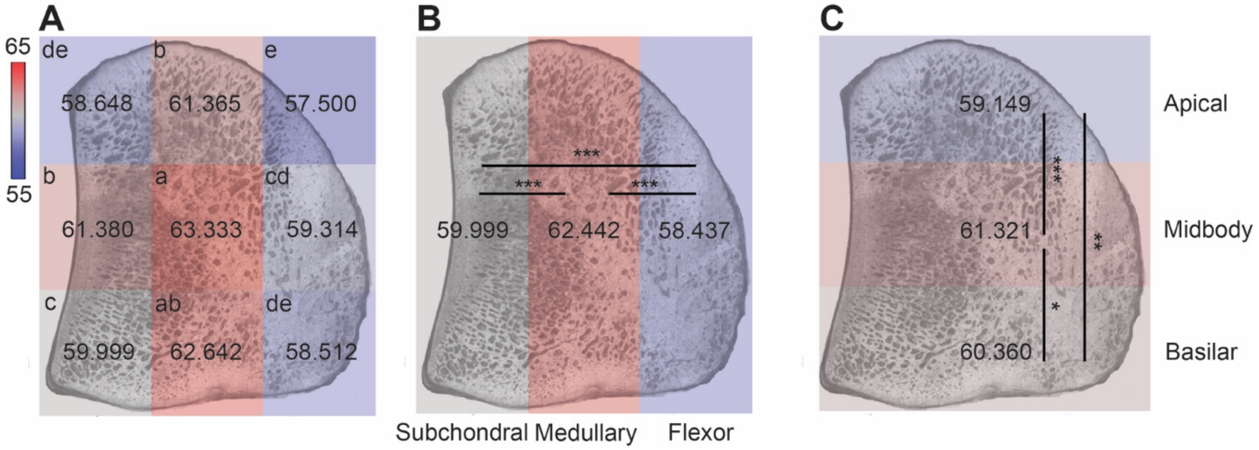

Figure S1. Regional comparison results within a mid-sagittal section of the proximal sesamoid bone of A) ash fraction across 3 dorsal-palmar regions; B) ash fraction across 3 proximal-distal regions; C) ash fraction across 9 anatomic sub-regions; (***: $p<0.001$; **: $p<0.01$; *: $p<0.05$: #: $0.05<p<0.1$). In 5C, there are significant differences across regions with no same letter.
